# Mechanical Properties of Latex-Modified Cement Stone under Uniaxial and Triaxial Cyclic Loading

**DOI:** 10.3390/polym16172543

**Published:** 2024-09-09

**Authors:** Qizhong Tian, Lianzhi Yang, Jie Zhang, Zhenzhong Xing

**Affiliations:** 1 Institute of Ocean Engineering and Technology, Ocean College, Zhejiang University, Zhoushan 316021, China; 12134111@zju.edu.cn; 2 Petroleum Engineering Technology Research Institute, Sinopec Shengli Oilfield Company, Dongying 257001, China; 3 School of Civil and Resource Engineering, University of Science and Technology Beijing, Beijing 100083, China; ylz_xiaozhu@126.com (L.Y.); m202310041@xs.ustb.edu.cn (Z.X.)

**Keywords:** cyclic loading, irreversible strain, well cement, hysteresis curve

## Abstract

During the cyclic injection and extraction process in underground storage wellbores, the cement sheath undergoes loading and unloading stress cycles. In this study, we investigated the mechanical properties of latex-modified cement stone (LMCS), widely used in oil and gas wells, through uniaxial and triaxial cyclic loading and unloading tests. The aim of the study was to determine the effect of various loading conditions on the compressive strength and stress–strain behavior of LMCS. The results show that the stress–strain curve of LMCS exhibits a hysteresis loop phenomenon, with the loop intervals decreasing throughout the entire cyclic loading and unloading process. As the number of cycles increases, the cumulative plastic strain of the LMCS increases approximately linearly. Under uniaxial cyclic loading and unloading conditions, the elastic modulus tends to stabilize. However, under triaxial conditions, the elastic modulus increases continuously as the number of cycles increases. This result provides data for engineering predictions. Furthermore, a comparison of the uniaxial and triaxial cyclic loading and unloading of LMCS shows that its cumulative plastic strain develops rapidly under uniaxial conditions, while the elastic modulus is larger under triaxial conditions. These findings provide a valuable reference for constructing underground storage wellbores.

## 1. Introduction

The growing demand for energy has heightened interest in underground storage technologies for renewable energy [1,2,3]. As reliance on these technologies increases, the structural integrity of underground storage wells has become a focal point of research [4,5]. In these wells, the cement sheath plays a crucial role during the cyclic injection and extraction processes, enduring a complex cycle of stress loading and unloading [6,7]. The safety and long-term reliability of the system are directly dependent on the stability of the cement sheath. As such, the deformation characteristics of the underground energy storage system under cyclic loading conditions provide deeper insights into the operational state and stability of cement within the system [8,9,10,11]. Additionally, understanding the accumulation pattern of plastic strain during cyclic loading helps in assessing the extent of deformation and damage over long-term operation, providing a reliable basis for operating the system safely. Latex can improve the flexibility, impermeability, and durability of cement. LMCS has significant advantages in oil and gas well engineering due to its anti-channeling, anti-corrosion, and mechanical properties [12,13,14]. Therefore, research into the cyclic loading performance of LMCS has significant theoretical and practical implications for developing and utilizing underground storage technologies.

Recently, researchers have extensively explored the mechanical properties, plastic strain accumulation, and damage evolution mechanisms of cement stone under cyclic loading and unloading conditions. Experiments have revealed the behavior of parameters, such as stress and strain, of cement paste under cyclic loading and unloading. Zhou et al. [8] conducted cyclic loading and unloading tests on cement stone at various temperatures and found that higher temperatures increased the cumulative residual strain in the cement stone, with the unloading curve exhibiting a distinct “rebound hysteresis” phenomenon. Zhang et al. [15] conducted triaxial compression and cyclic loading–unloading tests at different upper and lower deviatoric stresses. They analyzed the elastic modulus and Poisson’s ratio of cement stone under different deviatoric stress levels and found that plastic strain increases approximately linearly with increasing unloading strain. The existing literature provides little clarity about the mechanical properties of latex-modified cement stone (LMCS) under uniaxial and triaxial cyclic loading and unloading. Researchers, evidently, have rarely compared the mechanical performance of LMCS under these two conditions.

Some scholars have theoretically established a constitutive model for cement paste under cyclic loading and unloading. Cheng et al. [16] described the toughening and embrittlement reduction mechanisms of cement stone under dynamic loading from three aspects: strain dispersion effects, microcrack initiation and propagation effects, and interfacial delamination and tearing effects. Xu et al. [17] applied the minimum energy principle to describe crack development and evolution in cement stone under cyclic loading and unloading. However, no analysis was conducted on the changes in the loading method of cement stone with a fixed lower limit and variable upper limit of cyclic stress.

The plastic deformation and fatigue life of cement rings have also been examined under cyclic loading and unloading using numerical simulations. Yuan et al. [18] combined finite element analysis and experiments to predict the failure cycle number of cement sheaths under different temperatures and pressures. Zhang et al. [19] conducted a mechanical integrity analysis on the formation wellbore and cement sheath to determine the stress range for alternating injection and production. He et al. [20] developed a damage evolution model for cement stone, considering the effects of porosity and microcracks. This damage law was applied to the numerical analysis of the damage response during the service life of a gas storage cavern wellbore. However, the applicable conditions are restricted because of the limited analysis of the mechanical properties of cement stone under different cyclic stress limits. Hence, further research is needed into the development behavior of cumulative plastic strain and changes in parameters such as the strength and elastic modulus of LMCS. Deeper research into these areas will provide a theoretical reference and technical support for underground energy storage engineering.

In this study, the LMCS used is the current formula for Sinopec oil and gas well cement and has been widely applied in oil and gas well cements. To investigate the mechanical properties of LMCS under cyclic loading and unloading, uniaxial and triaxial cyclic tests were conducted. These tests evaluated the development of cumulative plastic strain and variations in parameters such as the elastic modulus under different cyclic conditions. The stress–strain curves, elastic modulus, and changes in compressive strength were analyzed for both loading scenarios. The findings offer scientifically validated recommendations for using LMCS in underground energy storage construction projects.

## 2. Experimental Program

### 2.1. Sample Preparation

The LMCS composition detailed in Table 1 includes cement, water, latex, stabilizer, latex defoamer, and defoamer. Latex decreases the elastic modulus of cement stone, which helps extend the lifespan of cement sheaths in oil and gas wells. Stabilizers play a crucial role in maintaining the uniformity and stability of the cement slurry system. Additionally, defoamers minimize bubbles in the slurry, thereby enhancing the strength of the cement stone. In compliance with the American Petroleum Institute’s API RP 10B-2-2013 standard [21] and the cement formula for Sinopec oil and gas wells, a water-to-cement ratio of 0.32 was selected. According to the Standard GB/T 19139-2012 [22], the cement slurry was prepared using a constant-speed mixer. The resulting stratified cement stone samples were placed in a curing box and exposed to a controlled environment with a constant temperature of 50 ± 5 °C and humidity of 95% ± 5%. After seven days in the curing box, the samples were restored to saturation and trimmed at the edges for a smooth finishing.

Each sample was assigned a systematic label following a specific format: “D number”, “DX number”, or “SX number,” where “D” represents the specimen used for uniaxial compression tests, “DX” represents samples designated for uniaxial cyclic loading and unloading tests, and “SX” represents triaxial cyclic loading and unloading tests. For example, a sample labeled “DX-2” indicates the second sample used for the uniaxial cyclic loading and unloading tests. 

### 2.2. Testing Methods

The uniaxial and triaxial cyclic loading and unloading tests were conducted using the TAW-1000 type electro-hydraulic servo-controlled cement stone testing system. This system is operated by two high-precision pumps and can withstand axial and confining pressures up to 1000 kN and 150 MPa, respectively. Accurate measurements of axial and radial strains were obtained using spring-loaded extensometers, and the entire testing process was managed using a dedicated computer.

**Figure 1:**
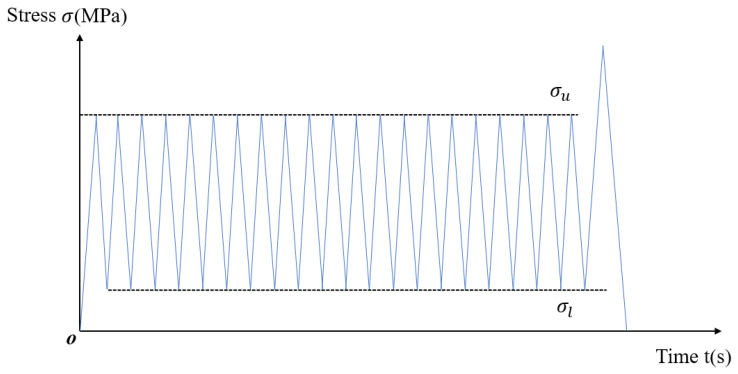
Load waveform curve.

The loading protocol designed for this experiment was based on the cyclic loading and unloading curve established by Zhou et al. [8]. As depicted in Figure 1, the test commenced by loading from zero to a predefined upper stress limit, followed by unloading to a set lower stress limit of 5 MPa, completing one cycle. In the figure, $\sigma_{u}$ represents the upper stress limit, while $\sigma_{l}$ indicates the lower stress limit. In order to prevent the test time from being too long and to explore the trend of plastic strain changes, each specimen was subjected to 20 cycles of loading and unloading. If the sample remained intact, loading continued until the sample fractured. The testing protocol utilized an axial displacement control mode with a constant loading rate of 0.05 mm/s. Data were recorded every 0.1 s throughout the experiment.

## 3. Experimental Results and Discussion

### 3.1. Uniaxial Cyclic Compression Tests

As shown in Table 2, uniaxial compression experiments were conducted on LMCS specimens to obtain their compressive strength, which was then used to establish the upper and lower limits of cyclic stress for the uniaxial cyclic compression tests. The uniaxial compression specimens are labeled D-1, D-2, and D-3, respectively.

The experimental conditions are presented in Table 3. The upper limit of cyclic stress refers to the stress conditions of different actual working environments. The upper stress limits were 12 MPa, 14 MPa, 16 MPa, 18 MPa, and 20 MPa, respectively. In the uniaxial cyclic loading and unloading tests, a lower stress threshold of 5 MPa was maintained for each experiment. Using axial displacement control, the loading rate was set at 0.05 mm/s. Each sample was subjected to 20 cycles of loading and unloading. If the sample did not fail after these cycles, loading continued until a complete stress–strain curve and peak strength data were obtained. These uniaxial cyclic loading and unloading tests were conducted on five different samples, labeled DX-1 through DX-5.

**Figure 2:**
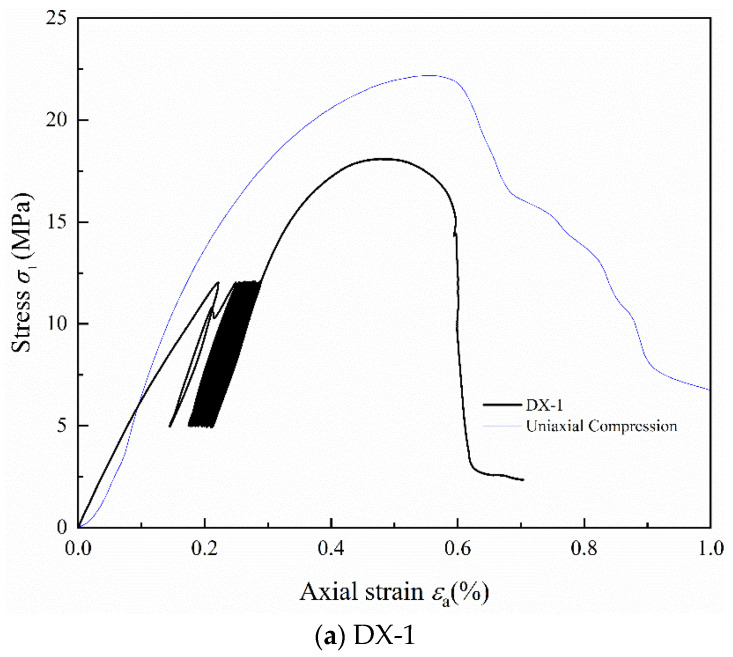

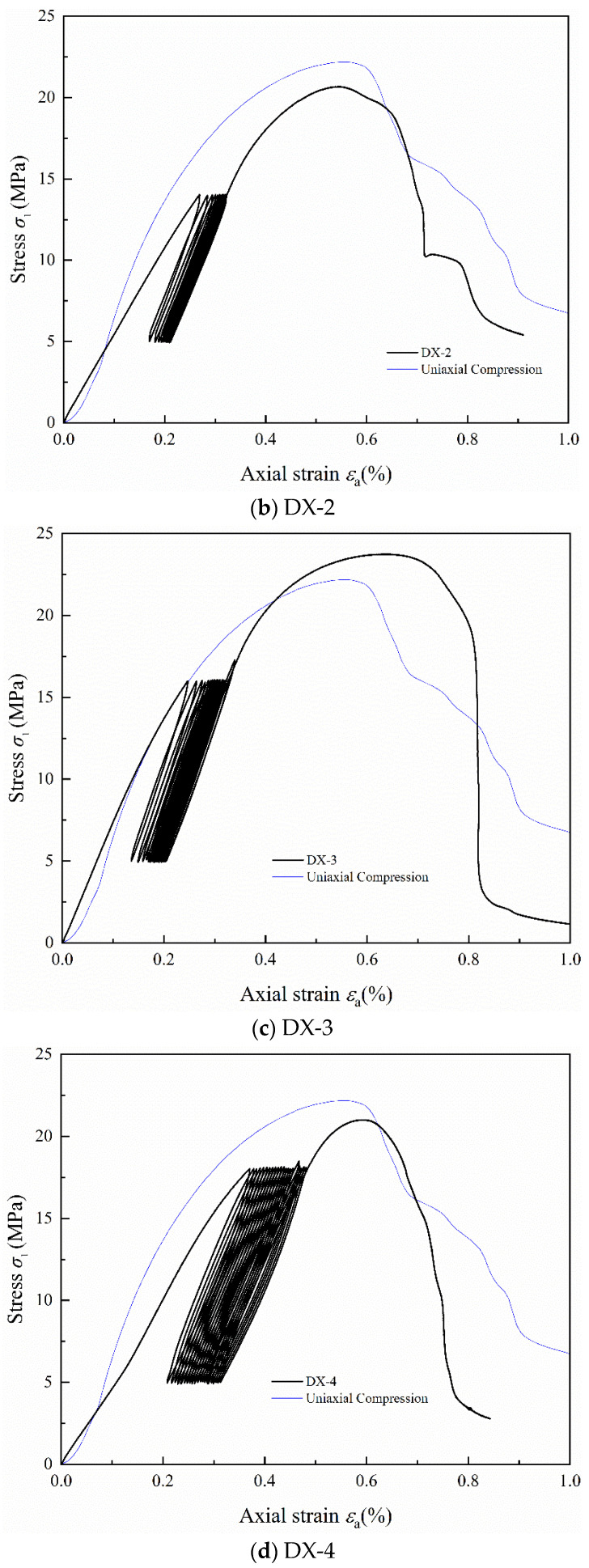

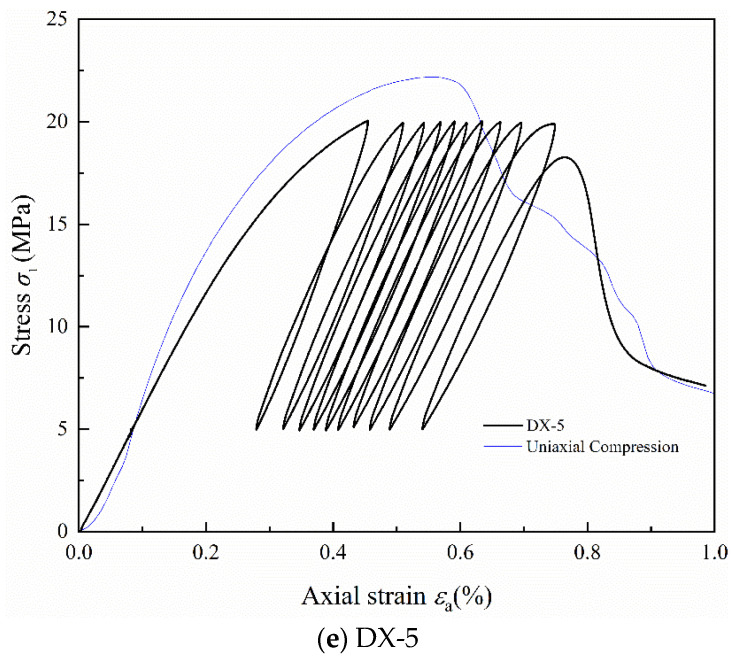
Stress–strain curves of LMCS under uniaxial cyclic loading and unloading.

Stress–strain curves for samples DX-1, DX-2, DX-3, DX-4, and DX-5 under cyclic loading and unloading are shown in Figure 2a, Figure 2b, Figure 2c, Figure 2d, and Figure 2e, respectively. The blue thin line in the figure represents the uniaxial compression stress–strain curve of LMCS. In the first loading cycle, the cement mortar sample initially undergoes compaction, which is followed by restoration. The concave, linear, and convex segments of the stress–strain curve represent the compaction, elastic, and plastic phases, respectively. The occurrence of plastic deformation in the cement stone indicates its distinct elasto-plastic characteristics. Upon reaching the upper stress limit, the sample is unloaded, producing a slightly downward convex unloading curve, which can be approximated as a straight line. When unloaded to 5 MPa, the curve does not return to its initial loading position, showing significant hysteresis, and the loading and unloading curves do not coincide, forming a “hysteresis loop.” The second cycle of loading and unloading continues, where the stress–strain curve of the cement stone follows a similar pattern of linear, then curved, changes during loading. When unloaded at the upper stress limit, the unloading curve approximates a straight line. Upon unloading to 5 MPa, the stress–strain curve shifts backward. As the cyclic loading and unloading continues, the stress–strain curve of the cement stone continually shifts backward, and plastic deformation accumulates. After the 20th loading and unloading cycle, the sample curve begins to rise continuously until the sample fails at the peak. In addition, as shown in Figure 2a, sample DX-1 shows a significant yield cross-section during the second loading cycle, which is not significant in other samples, possibly because of the compaction of large internal bubbles. Notably, sample DX-5 demonstrates a hysteresis curve density that alternates between loose to dense and settles as loose. This behavior is driven by the continuous opening and closing of microcracks during the cyclic loading process, which accelerates fatigue damage, culminating in failure during the 10th cycle. After the cyclic loading and unloading are completed, the axial strain of the specimens at failure ranges from 0.5% to 0.65%. All samples exhibited characteristics of brittleness, characterized by a rapid decrease in stress beyond the peak strength. The experimental data shows that the hysteresis curve interval gradually decreases. At lower stress levels, the curves are initially dense, but they become looser with the increase in the upper stress limits in the cyclic loading and unloading tests. By comparing the stress–strain curves of uniaxial compression and cyclic loading and unloading, it is evident that the peak strength of LMCS generally decreases after undergoing cyclic loading and unloading. Additionally, the peak strain shifts backward due to the cyclic loading and unloading process.

**Figure 3:**
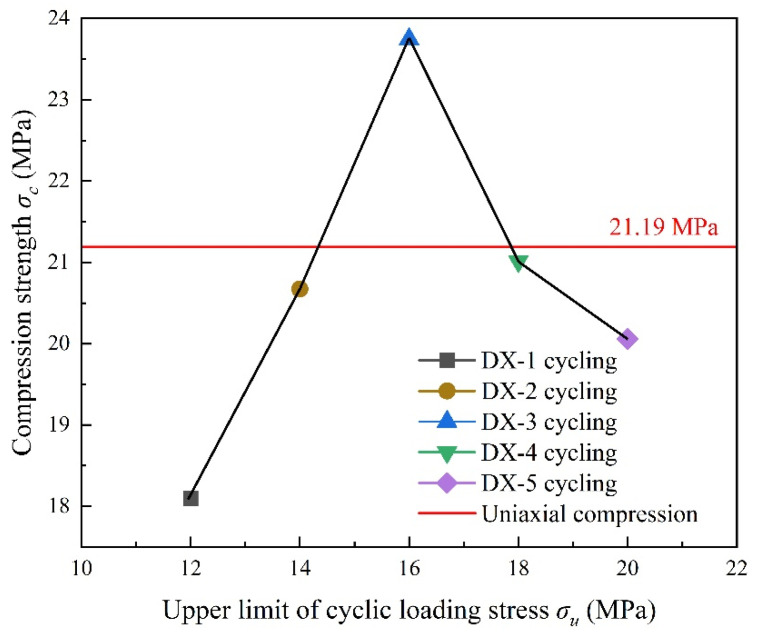
Relationship between compressive strength and upper limit of uniaxial cyclic compressive stress.

Table 4 presents the results of the LMCS uniaxial cyclic compression tests. Figure 3 illustrates the relationship between the compressive strength of LMCS and the upper stress limits of uniaxial cyclic compression. The red line in Figure 3 represents the average compressive strength results from these uniaxial compression tests on LMCS. Except for specimen DX-3, the compressive strength decreases after cyclic loading and unloading. This decrease might be due to the continuous opening and closing of cracks in the samples during the test, which lowers the damage threshold of LMCS. This result is consistent with previous findings [23,24]. The higher compressive strength at an upper stress limit of 16 MPa, compared to uniaxial compression, could be due to layering in the cement slurry during curing, resulting in samples with a higher cement content. The mechanical performance of LMCS after cyclic loading and unloading under different loading conditions can provide reference for the design of loading conditions under actual working conditions.

Generally, the total deformation of cement stone under cyclic loading is divided into elastic and plastic strain components, as detailed by Peng et al. [25] and Su et al. [26]. For LMCS, the plastic strain is defined as the cumulative plastic strain accumulated each time the material is loaded and unloaded up to the upper stress limit. The plastic strain of LMCS can be calculated using Equation (1) [27,28].

(1) $$\varepsilon_{p}=\ln(1+\varepsilon_{a})-\frac{\sigma_{1}(1+\varepsilon_{a})}{E},$$

where $\varepsilon_{a}$ is axial strain, $\varepsilon_{p}$ is cumulative plastic strain, $\sigma_{1}$ is axial stress, and E is elastic modulus.

**Figure 4:**
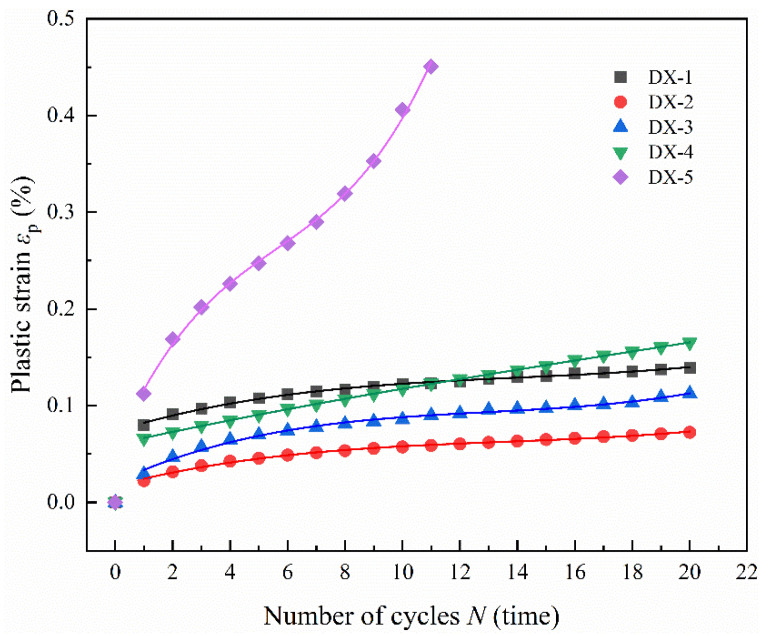
Relationship between cumulative plastic strain and the number of uniaxial cyclic compression cycles.

Sample DX-5 failed after 11 cycles of loading, whereas the other samples persisted for 20 cycles. Figure 4 illustrates the relationship between the cumulative plastic strain and the number of cycles under varying upper stress limits for LMCS in uniaxial cyclic loading tests. As the data show, the cumulative plastic strain for DX-1, DX-2, DX-3, and DX-4 typically increases linearly as the number of cycles increases. Notably, the plastic strain surges most significantly after the first cycle and, to a lesser extent, after the second cycle. With each subsequent cycle, the increment in cumulative plastic strain stabilizes, peaking after the 20th cycle. The cumulative plastic strain in the cement stone consistently increases throughout the entire cyclic loading and unloading process. After 20 cycles, the cumulative plastic strain for DX-1, DX-2, DX-3, and DX-4 remains below 0.15%. However, in the final two cycles, sample DX-5 experiences a significant increase in plastic strain, and failure occurs at a cumulative plastic strain of 0.45%, indicating an acceleration in the accumulation of plastic strain in LMCS during the two cycles prior to fatigue failure. Overall, the plastic strain accumulation in LMCS before fatigue failure has a fast–slow–fast trend [29].

Based on the relationship obtained between the cumulative plastic strain of LMCS and the number of cycles, a polynomial function was fitted to predict the development of the cumulative plastic strain in LMCS. To estimate the plastic strain growth more accurately, the effect of the zero point before cycling begins was ignored when fitting the function. $R^{2}$ is used to represent the goodness of curve fitting. $R^{2}$ can be calculated as follows:

(2) $$R^{2}=1-\frac{SS_{res}}{SS_{tot}}$$

where $SS_{res}$ denotes the sum of the squares of the residuals and $SS_{tot}$ indicates the total sum of squares.

As shown in Table 5, the cumulative plastic strain curves for LMCS were obtained for various operating conditions. The curves were then imported into numerical simulation software to model and analyze the materials’ damage and lifespan under repeated stress and strain.

**Figure 5:**
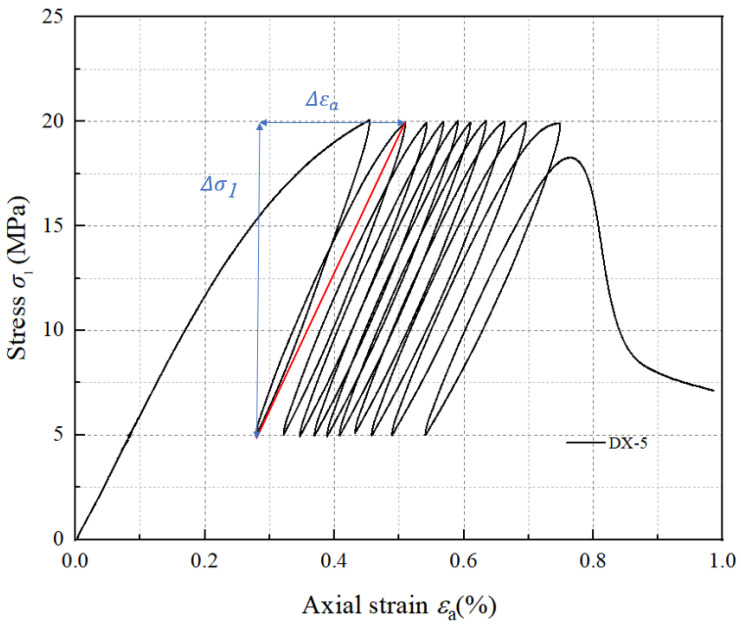
Calculation method for secant elastic modulus.

**Figure 6:**
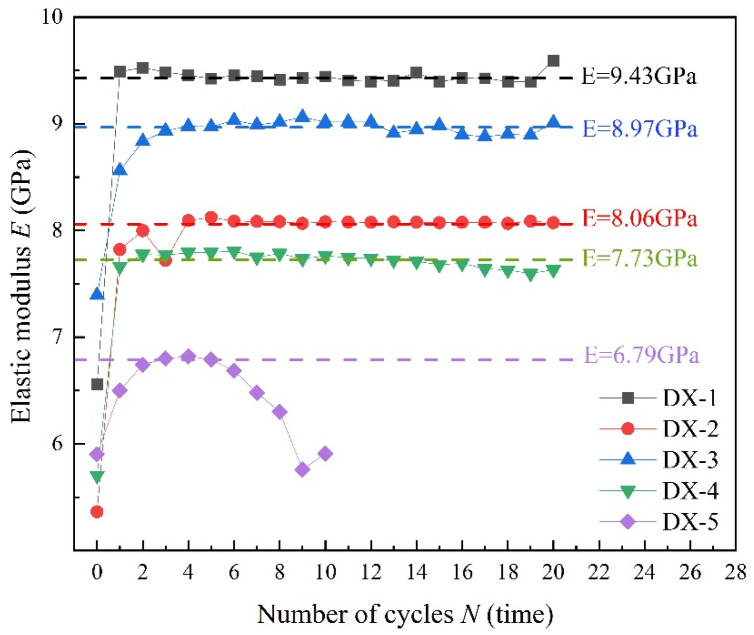
Evolution of elastic modulus with number of cycles under uniaxial cyclic compression tests.

As shown in Figure 5, we define the slope between the upper stress limit point and the lower stress limit point during loading and unloading as the secant elastic modulus [30]. The elastic modulus is calculated using Equation (3):

(3) $$E=\Delta \sigma_{1}/\Delta \varepsilon_{a},$$

where $\Delta \sigma_{1}$ and $\Delta \varepsilon_{a}$ are the differences in stress and strain between the lowest point of unloading in the previous cycle and the highest point of loading in the next cycle, respectively. The slope of the red line in Figure 5 is equal to the magnitude of the elastic modulus. Accordingly, Figure 6 illustrates the evolution of the elastic modulus of five samples under different upper stress limits in uniaxial cyclic loading and unloading. When LMCS undergoes cyclic loading and unloading, its elastic modulus significantly increases after the first cycle and then gradually stabilizes. The increase in elastic modulus minimizes the samples’ deformation, indicating that LMCS hardens during cyclic loading and unloading. This process of continuous plastic strain accumulation corresponds to the changes in plastic strain described earlier. These findings are consistent with previous tests results [15,31,32]. After entering the hardening cycle, the elastic modulus of LMCS can reach 7–10 GPa. For a more effective representation of the changes in the elastic modulus of LMCS during cyclic loading and unloading, the elastic modulus of LMCS is simplified to two values using numerical calculations. The first value is used before the initial loading and unloading cycle is completed, and the second value is used thereafter. Unlike the other samples, sample DX-5 shows a noticeable decrease in elastic modulus after an initial increase, which drops below the initial modulus in the cycles prior to failure. This phenomenon occurs several cycles before failure, indicating that LMCS gradually softens before experiencing fatigue failure, eventually entering a softening phase post failure.

### 3.2. Triaxial Cyclic Compression Tests

The compressive strength of LMCS under a confining pressure of 5 MPa is 36.25 MPa. In the triaxial cyclic loading and unloading tests, all experiments were conducted at this confining pressure, which also served as the lower stress limit. The upper stress limits were chosen based on geological stress measurements at various depths. As detailed in Table 6, these upper limits were set at 16 MPa, 18 MPa, 23 MPa, 27 MPa, and 33 MPa. The loading method employed axial displacement control at a rate of 0.05 mm/s. Each sample underwent 20 cycles of loading and unloading. If a sample did not fail, loading continued to produce comprehensive stress–strain curves and determine peak strength. Five triaxial cyclic loading and unloading tests were performed on distinct samples, labeled SX-1 through SX-5.

**Figure 7:**
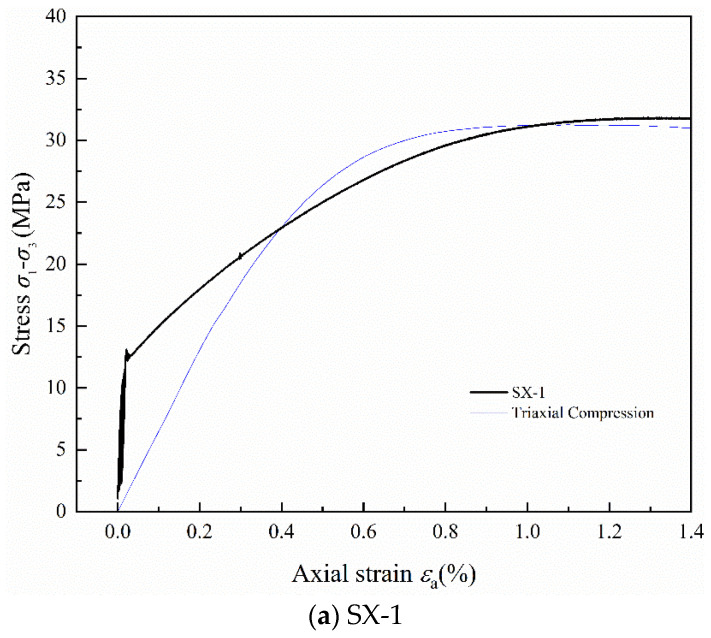

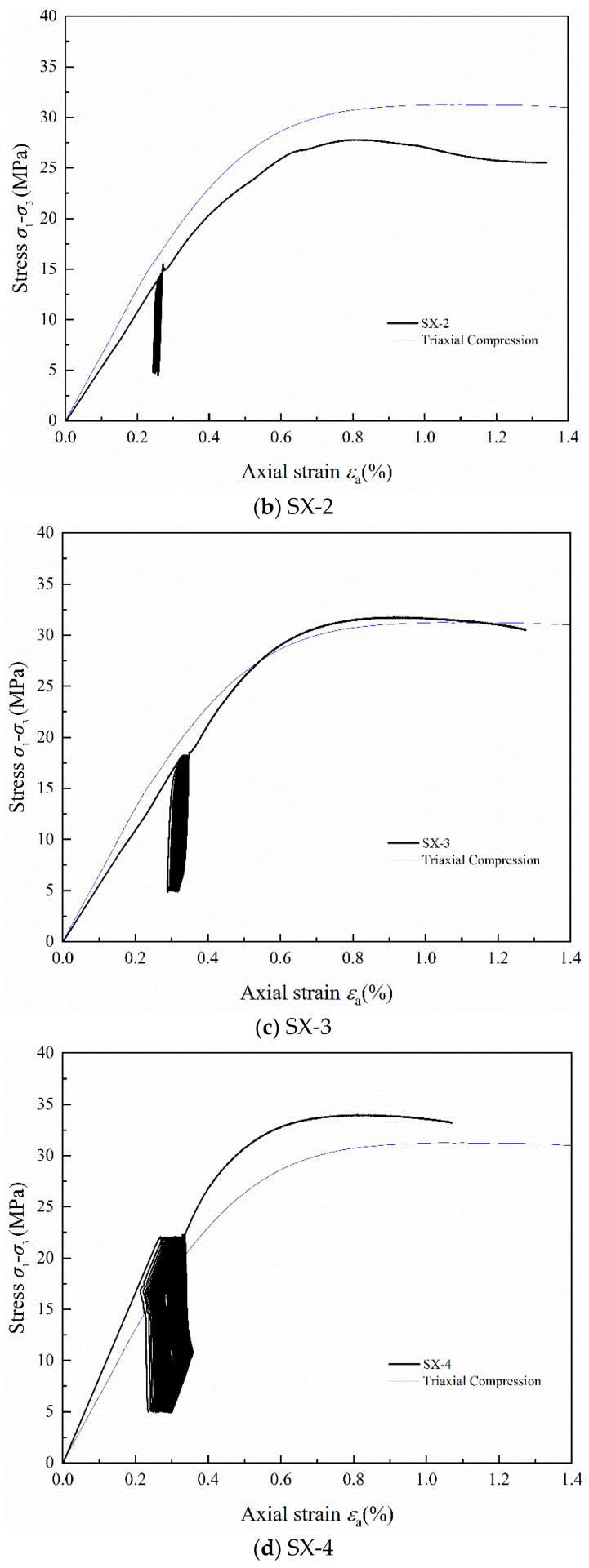

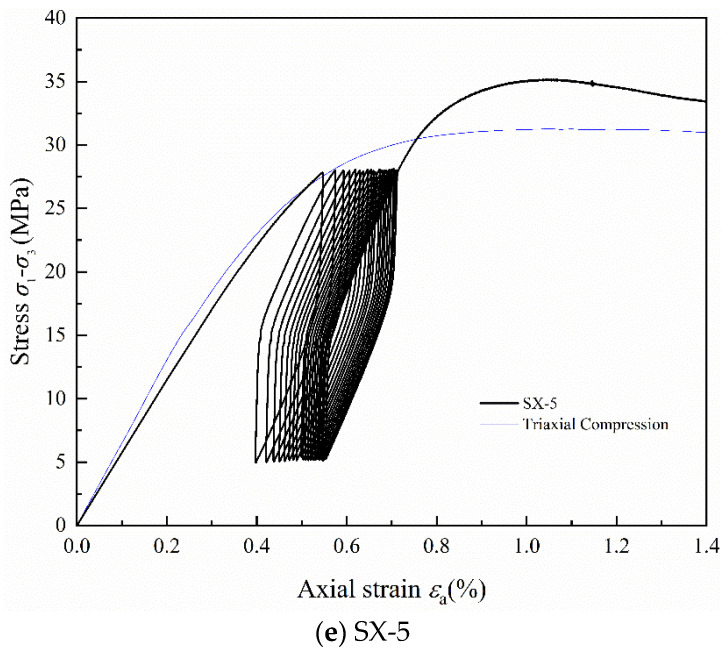
Stress–strain curves of LMCS under triaxial cyclic loading and unloading.

Figure 7 shows the stress–strain curves of LMCS under triaxial cyclic loading and unloading conditions. The thin blue line in the figure represents the triaxial compression stress–strain curve of LMCS. From comparison with Figure 2, it can be seen that during the loading phase and the linear elastic compression stage, the behavior of the triaxial cyclic samples is similar to that of the uniaxial cyclic samples. However, aside from specimen SX-5, the stress–strain curve prior to the onset of cyclic loading and unloading remains almost entirely linear. Thus, the selected upper stress limit keeps the samples within their elastic range. Moreover, during triaxial cyclic loading and unloading, LMCS exhibits a more stable behavior in terms of peak strain softening across the yielding and post-peak stages. When peak stress is reached, the triaxial compressed samples enter a plateau phase, exhibiting material properties closer to those of ductile materials. The hysteresis curves of SX-1 and SX-5 are very dense because of their lower upper stress limits. The hysteresis loops of SX-3 and SX-4 are more pronounced than those of other curves because of their higher upper stress limits, creating more room for the hysteresis loops to form in a distinctive quadrilateral shape unique to triaxial cyclic loading and unloading [30]. Comparing the stress–strain curves of triaxial compression with cyclic loading and unloading, it is evident that the peak strain of the samples shifts slightly backward after undergoing cyclic loading and unloading.

The results of the triaxial cyclic loading and unloading tests for the cement stone samples are shown in Table 7. The compressive strength of the samples under various upper stress limits during cyclic loading and unloading ranges from 32.81 MPa to 40.16 MPa, with an average of 37.53 MPa.

**Figure 8:**
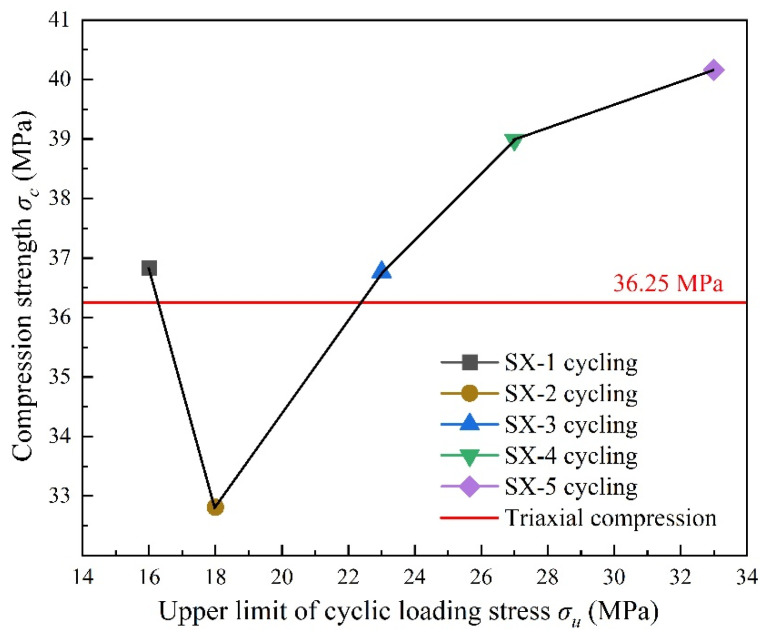
Relationship between compressive strength and upper limit of triaxial cyclic compressive stress.

Figure 8 displays the relationship between the compressive strength of LMCS and the upper stress limits of triaxial cyclic compression. The red line represents the average compressive strength results from the triaxial compression tests on LMCS at a confining pressure of 5 MPa. It can be observed that, except for sample SX-2, the compressive strength increases after cyclic loading and unloading. This increase may be due to the triaxial cyclic load raising the damage threshold of LMCS, increasing the compactness and strength of the samples. The lower compressive strength of SX-2, compared to the triaxial compression, could be due to the layering in the cement slurry during curing, lowering the cement content of the sample. The compressive strength of LMCS after triaxial cyclic loading and unloading can provide a reference for designing loading conditions based on actual working conditions.

**Figure 9:**
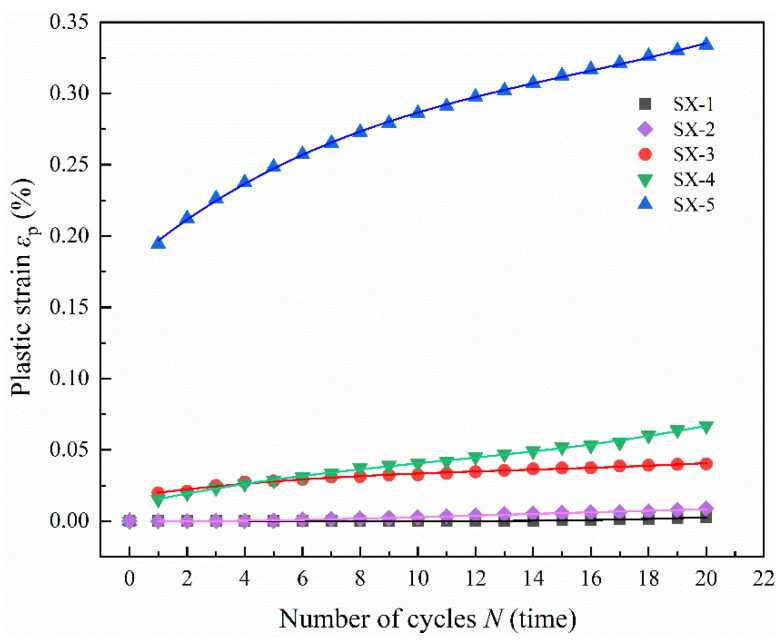
Relationship between cumulative plastic strain and the number of triaxial cyclic compression cycles.

All samples undergoing triaxial cyclic loading and unloading were subjected to 20 cycles of loading. Figure 9 illustrates the relationship between cumulative plastic strain and the number of cyclic loads for LMCS under varying upper stress limits of triaxial cyclic loading. Samples SX-3 and SX-4 exhibit behavior similar to that seen in uniaxial cyclic loading and unloading. The cumulative plastic strain for these samples exhibits an approximately linear growth as the number of cycles increases. The largest increase in plastic strain occurs after the first cycle, with a smaller increase following the second cycle, and subsequent increments tend to stabilize with each additional cycle of loading and unloading, peaking after the 20th cycle. Samples SX-1 and SX-2 exhibit no plastic strain in the initial cycles because the upper stress limits of the cycles are far below the yield stress of LMCS at a confining pressure of 5 MPa. However, as the cyclic loading and unloading progresses, SX-1 begins to exhibit plastic strain from the 14th cycle, and SX-2 from the 4th cycle, with both showing rapid increases thereafter. The rate of plastic strain accumulation is a critical factor for determining the fatigue life of the samples, where, typically, a slower accumulation indicates a longer lifespan. The observed strain accumulation curves suggest that SX-1 and SX-5 are likely to withstand more cycles before reaching fatigue failure compared to the other samples.

A polynomial function was used to model the relationship between the cumulative plastic strain of LMCS and the number of triaxial cyclic loading and unloading cycles. In Figure 9, the various colored lines depict the curves generated by the plastic strain fitting functions for each specimen. Table 8 shows a strong correlation in the high R-squared values between the fitted curves and the experimental data points, indicating a good fit. This modeling of cumulative plastic strain under triaxial cyclic loading and unloading conditions is crucial for understanding interfacial detachment issues. Such detachments can occur because of the inconsistent deformations between the cement sheath and the casing or formation interfaces in energy storage wellbores. An accurate prediction of the development of cumulative plastic strain can help engineers better assess and mitigate potential risks associated with interface integrity in these critical structures.

**Figure 10:**
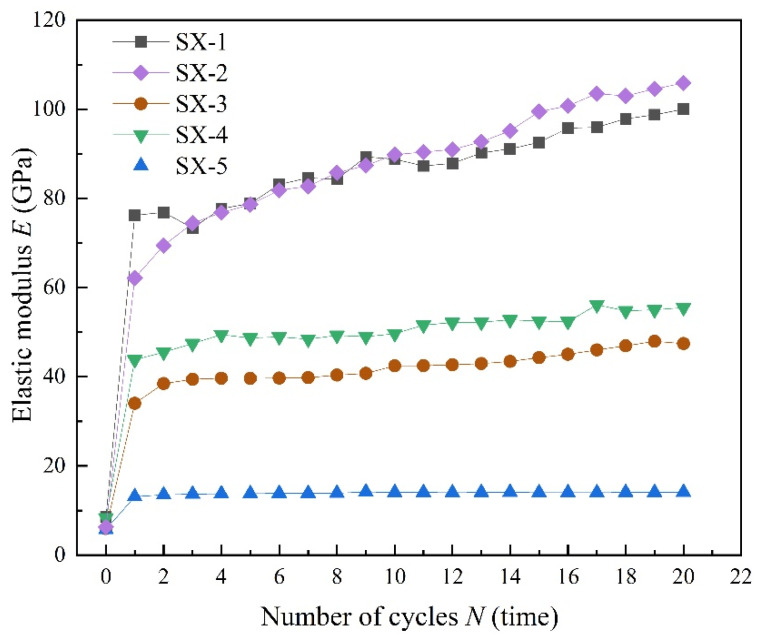
Evolution of elastic modulus with number of cycles under triaxial cyclic compression tests.

Figure 10 illustrates the progression of the elastic modulus in five samples subjected to different upper stress limits during triaxial cyclic loading and unloading. These data echo observations from uniaxial cyclic loading and unloading, where the elastic modulus of LMCS exhibits a significant increase after the initial cycle. However, a key difference in the triaxial setup is that the elastic modulus tends to fluctuate over successive cycles, despite its overall increasing trend. This behavior suggests that LMCS undergoes continuous hardening throughout the triaxial cyclic loading and unloading process [33,34,35]. After entering the triaxial hardening cycle, the secant elastic modulus can reach 40–100 GPa. This is because under triaxial compression, the upper stress limit of the cycles is far below the yield stress of LMCS, resulting in very small axial strain increments in each cycle. Therefore, the calculated secant elastic modulus is very high. The initial elastic modulus of samples SX-1 to SX-5 is relatively similar, ranging between 5 and 10 GPa. Compared to uniaxial cyclic loading and unloading, the overall elastic modulus of LMCS increases more substantially. For an effective modeling of the observed trend and for a simplified calculation, the elastic modulus of the cement sheath is made to increase linearly with each additional loading cycle. In this approach, the initial and final values of the elastic modulus are used, which are derived from the experimental data under specific operational conditions. The linear configuration helps in streamlining numerical simulations, allowing for more accurate predictions of the cement sheath’s behavior under cyclic loading conditions. Aligning the model closely with empirical data ensures that the simulations are both realistic and technically sound, facilitating better decision-making in engineering applications.

### 3.3. Discussion on the Difference between Uniaxial and Triaxial Cyclic Compressions

**Figure 11:**
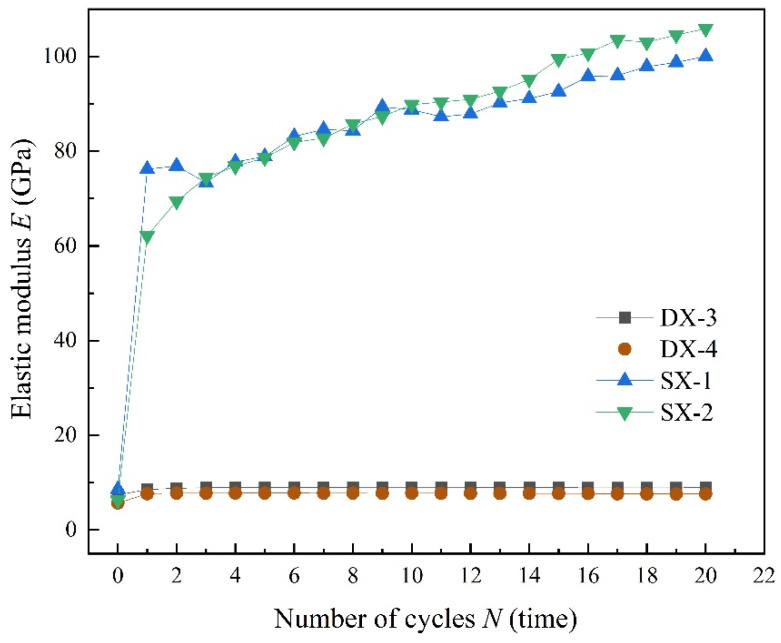
Evolution of elastic modulus under the same upper limit of cyclic stress.

Figure 11 shows that when comparing changes in the elastic modulus of specimens subjected to both uniaxial and triaxial cyclic loading and unloading under the same cyclic stress limits, the elastic modulus of LMCS is consistently higher under the triaxial conditions. The maximum cyclic stress for specimens DX-3 and SX-1 is set at 16 MPa, while for specimens DX-4 and SX-2, it is set at 18 MPa. Moreover, as the cyclic loading progresses, triaxial cyclic loading causes a gradual increase in the elastic modulus of LMCS, thereby increasing the difference in elastic modulus between the two.

**Figure 12:**
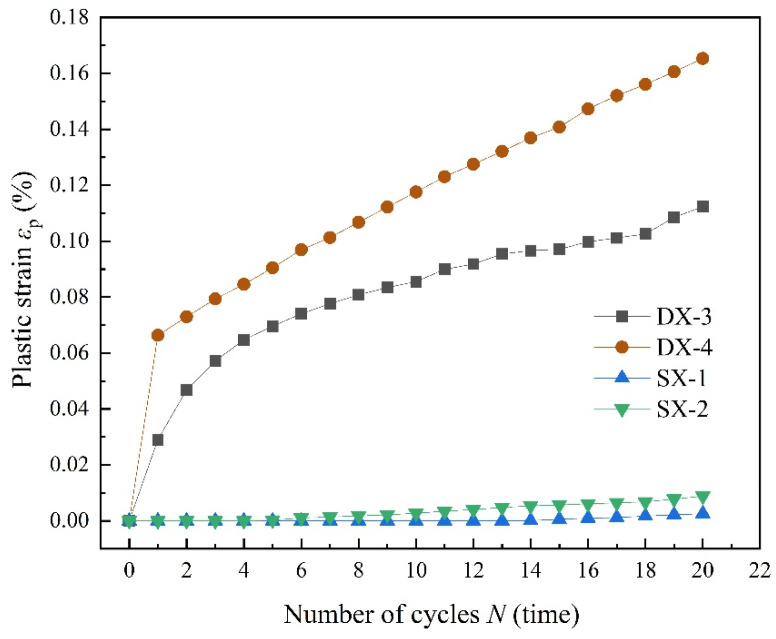
Evolution of cumulative plastic strain under the same upper limit of cyclic stress.

Figure 12 illustrates a comparison between the development of cumulative plastic strain and changes in elastic modulus for specimens subjected to uniaxial and triaxial cyclic loading and unloading under identical cyclic stress limits. The figure shows that the rate of cumulative plastic strain growth in LMCS is considerably higher under uniaxial conditions, suggesting that LMCS is more prone to early fatigue failure when subjected to uniaxial cyclic loading and unloading. This behavior can be attributed to the uniaxial conditions focusing stress along a single axis, thereby intensifying the material’s deformation and accelerating the fatigue process.

## 4. Conclusions

In this study, we comprehensively investigated the mechanical behavior of LMCS under various loading and unloading scenarios, conducting both uniaxial and triaxial cyclic tests. The conclusions drawn are as follows:

(1) The peak strength of LMCS is generally lower under uniaxial cyclic loading compared to monotonic loading, whereas it is typically higher under triaxial cyclic loading. This increase may be attributed to the triaxial cyclic load, which raises the damage threshold of LMCS, thereby increasing the compactness and strength of the samples.
(2) Under cyclic loading and unloading conditions, the hysteresis loop interval of the stress–strain curve of LMCS gradually decreases. The stress level is negatively correlated with the density of the hysteresis curve.
(3) As the number of cycles increases, the accumulated plastic strain in LMCS increases linearly.
(4) The cyclic loading and unloading process fundamentally acts as a hardening mechanism for LMCS. Notably, after the initial cycle, the elastic modulus of LMCS increases significantly but tends to stabilize when subjected to uniaxial cyclic loading and unloading. Conversely, under triaxial cyclic loading and unloading, the elastic modulus does not stabilize; it continues to increase with each successive cycle.
(5) A comparison of uniaxial and triaxial cyclic loading and unloading of LMCS shows that cumulative plastic strain develops rapidly under uniaxial conditions, while the elastic modulus is larger under triaxial conditions. This result can provide reference for engineering life prediction and numerical modeling analysis.
(6) The findings of this study can provide a reference and guidance for the cyclic injection and extraction engineering of underground storage wellbores.

## Figures and Tables

**Table 1:** Mix proportions of latex-modified cement.

Raw Material	Cement	Water	KCM028 (Latex)	KCM018A (Stabilizer)	KCM043 (Latex Defoamer)	KCM003 (Defoamer)
Proportion (%)	70.18	22.69	6.33	0.25	0.38	0.17

**Table 2:** Uniaxial compression test results of cement stone specimens.

Specimen Number	Length *L* (mm)	Diameter *D* (mm)	Density *ρ* (g/cm^3^)	Compressive Strength *σ_c_* (MPa)
D-1	50.54	24.62	1.90	22.21
D-2	49.58	24.30	1.90	22.09
D-3	50.80	24.32	1.89	19.28
Mean value	50.31	24.41	1.90	21.19

**Table 3:** Uniaxial cyclic loading and unloading test conditions for cement stone specimens.

Specimen Number	Length *L* (mm)	Diameter *D* (mm)	Density *ρ* (g/cm^3^)	Upper Limit of Stress *σ_u_* (MPa)	Lower Limit of Stress *σ_L_* (MPa)
DX-1	50.61	24.44	1.85	12.0	5.0
DX-2	50.33	24.33	1.88	14.0	5.0
DX-3	51.01	24.71	1.90	16.0	5.0
DX-4	49.24	24.49	1.89	18.0	5.0
DX-5	49.72	24.60	1.89	20.0	5.0
Mean value	50.18	24.51	1.88	16.0	5.0

**Table 4:** Uniaxial cyclic loading and unloading test results of cement stone specimens.

Specimen Number	Number of Cycles N (Time)	Compressive Strength *σ_c_* (MPa)
DX-1	20	18.10
DX-2	20	20.67
DX-3	20	23.75
DX-4	20	21.01
DX-5 (Damaged)	11	20.06
Mean value	18.2	20.72

**Table 5:** Variation law of plastic strain under uniaxial cyclic loading and unloading.

Specimen Number	Plastic Strain Formula	*R* ^2^
DX-1	$\varepsilon_{p}=1.29\times 10^{-5}N^{3}-0.00055N^{2}+0.00905N+0.07371$	0.99
DX-2	$\varepsilon_{p}=1.19\times 10^{-5}N^{3}-0.00049N^{2}+0.00773N+0.01718$	0.99
DX-3	$\varepsilon_{p}=2.41\times 10^{-5}N^{3}-0.00093N^{2}+0.01365N+0.02085$	0.99
DX-4	$\varepsilon_{p}=1.85\times 10^{-6}N^{3}-0.0001N^{2}+0.00658N+0.06009$	0.99
DX-5	$\varepsilon_{p}=5.19\times 10^{-4}N^{3}-0.0087N^{2}+0.06931N+0.05534$	0.99

**Table 6:** Triaxial cyclic loading and unloading test conditions for cement stone specimens.

Specimen Number	Length *L* (mm)	Diameter *D* (mm)	Density *ρ* (g/cm^3^)	Confining Pressure *σ*_3_ (MPa)	Upper Limit of Stress *σ_u_* (MPa)	Lower Limit of Stress *σ_L_* (MPa)
SX-1	49.36	24.51	1.89	5.0	17.0	5.0
SX-2	50.18	24.54	1.84	5.0	18.0	5.0
SX-3	51.03	24.26	1.89	5.0	23.0	5.0
SX-4	50.72	24.30	1.88	5.0	27.0	5.0
SX-5	49.60	24.60	1.90	5.0	33.0	5.0
Mean value	50.18	24.44	1.88	5.0	23.8	5.0

**Table 7:** Triaxial cyclic loading and unloading test results of cement stone specimens.

Specimen Number	Number of Cycles N (Time)	Compressive Strength *σ_c_* (MPa)
SX-1	20	36.83
SX-2	20	32.81
SX-3	20	36.76
SX-4	20	38.98
SX-5	20	40.16
Mean value	20	37.53

**Table 8:** Variation law of plastic strain under triaxial cyclic loading and unloading.

Specimen Number	Plastic Strain Formula	*R* ^2^
SX-1	$\varepsilon_{p}=1.9\times 10^{-6}N^{3}-3.7\times 10^{-6}N^{2}+1.09\times 10^{-4}N-0.00012$	0.98
SX-2	$\varepsilon_{p}=7.8\times 10^{-8}N^{3}+6\times 10^{-7}N^{2}+4.8\times 10^{-4}N-0.00203$	0.99
SX-3	$\varepsilon_{p}=3.7\times 10^{-6}N^{3}-1.5\times 10^{-4}N^{2}+2.79\times 10^{-3}N+0.00173$	0.99
SX-4	$\varepsilon_{p}=7.6\times 10^{-6}N^{3}-2.4\times 10^{-4}N^{2}+4.63\times 10^{-3}N+0.01102$	0.99
SX-5	$\varepsilon_{p}=1.7\times 10^{-5}N^{3}-8.1\times 10^{-4}N^{2}+1.69\times 10^{-3}N+0.18071$	0.99

## Data Availability

The original contributions presented in the study are included in the article, further inquiries can be directed to the corresponding author.

## References

[1] Wang Y., Wen Z., Xu M., Kosajan V. (2024). The carbon-energy-water nexus of the carbon capture, utilization, and storage technology deployment schemes: A case study in China’s cement industry. Appl. Energy.

[2] Wang Z., Lyu X., Shi W., Feng X.T., Qiao L., Kong R. (2024). Assessing the effect of intermediate principal geostress on the caprock integrity for underground gas storage. Gas Sci. Eng..

[3] Shoushtari S., Namdar H., Jafari A. (2023). Utilization of CO_2_ and N_2_ as cushion gas in underground gas storage process: A review. J. Energy Storage.

[4] Moradi P., Chahardowli M., Simjoo M. (2023). Insights into underground gas storage in water-wet carbonate saline aquifers: The use of fluorinated surfactants to change the wettability. Fuel.

[5] Long K., Tang Y., He Y., Luo Y., Hong Y., Sun Y., Rui Z. (2024). Full-cycle enhancing condensate recovery-underground gas storage by integrating cyclic gas flooding and storage from gas condensate reservoirs. Energy.

[6] Yang H., Bu Y., Guo S., Liu H., Du J., Cao X. (2021). Effects of in-situ stress and elastic parameters of cement sheath in salt rock formation of underground gas storage on seal integrity of cement sheath. Eng. Fail. Anal..

[7] Patel H., Salehi S. (2021). Structural integrity of liner cement in oil & gas wells: Parametric study, sensitivity analysis, and risk assessment. Eng. Fail. Anal..

[8] Zhou S., Liu R., Zeng H., Zeng Y., Zhang L., Zhang J., Li X. (2019). Mechanical characteristics of well cement under cyclic loading and its influence on the integrity of shale gas wellbores. Fuel.

[9] Wang X., Xu M., Qin Y., Song J., Chen R., Yin Z. (2023). The Effect of Polymer Elastic Particles Modified with Nano-Silica on the Mechanical Properties of Oil Well Cement-Based Composite Materials. Polymers.

[10] Aluah R., Oni O., Fadairo A., Pothana P. (2024). Enhancing the performance of class G cement for subsurface gas storage and well completion: Synergistic impact of North Dakota’s fly ash and eggshell powder. Powder Technol..

[11] Yan R., Wang L., Ni Y., Zhang S., He Z., Guan B. (2024). A Study on the Properties of Composite Modified Mortar with Styrene–Butadiene Rubber Latex and Silica Fume. Polymers.

[12] Xie Z., Yuan Q., Yao H., Zhong F., Jiang M. (2023). Hardened properties and microstructure change of sulphoaluminate cement modified with different doses of styrene-butadiene rubber latex. Constr. Build. Mater..

[13] Lu Z., Kong X., Zhang Q., Cai Y., Zhang Y., Wang Z., Dong B., Xing F. (2016). Influences of styrene-acrylate latexes on cement hydration in oil well cement system at different temperatures. Colloids Surf. A Physicochem. Eng. Asp..

[14] Hoy M., Tran N.Q., Suddeepong A., Horpibulsuk S., Mobkrathok M., Chinkulkijniwat A., Arulrajah A. (2023). Improved fatigue properties of cement-stabilized recycled materials–Lateritic soil using natural rubber latex for sustainable pavement applications. Transp. Geotech..

[15] Zhang T., Xu W., Wang R., Yan L., He M. (2021). Deformation characteristics of cement mortar under triaxial cyclic loading: An experimental investigation. Int. J. Fatigue.

[16] Cheng X., Chen Z., Gu T., Zeng L., Yao L., Chen Z., Huang K., Zhang Z., Zhang C., Liu K. (2021). Study on the dynamic and static mechanical properties of microsphere rubber powder reinforced oil well cement composites. Constr. Build. Mater..

[17] Xu Y., Yang R., Chen P., Ge J., Liu J., Xie H. (2022). Experimental study on energy and failure characteristics of rubber-cement composite short-column under cyclic loading. Case Stud. Constr. Mater..

[18] Yuan Z., Teodoriu C., Schubert J. (2013). Low cycle cement fatigue experimental study and the effect on HPHT well integrity. J. Pet. Sci. Eng..

[19] Zhang S., Yan Y., Sheng Z., Yan X. (2021). Uncertainty failure risk quantitative assessments for underground gas storage near-wellbore area. J. Energy Storage.

[20] He T., Wang T., Xie D., Daemen J.J.K. (2022). The mechanism of pores enhancing the deformation of completion cement under confining pressure. Cem. Concr. Compos..

[21] (2012). Test Method for Oil Well Cement.

[22] (2013). Recommended Practice for Testing Well Cements.

[23] Miao S., Pan P.Z., Hou W., He B., Yu P. (2022). Stress intensity factor evolution considering fracture process zone development of granite under monotonic and stepwise cyclic loading. Eng. Fract. Mech..

[24] Shu R., Kong L., Li T., Jian T., Zhou Z. (2023). Accumulated plastic deformation behavior of granite residual soil under cyclic loading considering the influence of initial unloading. Transp. Geotech..

[25] Peng K., Zhou J., Zou Q., Yan F. (2019). Deformation characteristics of sandstones during cyclic loading and unloading with varying lower limits of stress under different confining pressures. Int. J. Fatigue.

[26] Su C., Zhu H., Cai W., Wu W., Zhang Q. (2023). Elastic perfect plastic rock mass analysis and equivalent GSI determination considering strain-softening behavior and three-dimensional strength. Tunn. Undergr. Space Technol..

[27] Zhong J., Xu T., Wang W., Guan K., Song M., Huang S., Zhang S. (2021). Use of database and small punch test to estimate true stress-plastic strain curve of steels. Int. J. Press. Vessel. Pip..

[28] Xi Y., Li J., Tao Q., Guo B., Liu G. (2020). Experimental and numerical investigations of accumulated plastic deformation in cement sheath during multistage fracturing in shale gas wells. J. Pet. Sci. Eng..

[29] Liu H., Pei J., Liu J., Xiao M., Zhuo L., Xie H. (2023). Influence of volume compression on the unloading deformation behavior of red sandstone under damage-controlled cyclic triaxial loading. J. Rock Mech. Geotech. Eng..

[30] Peellage W.H., Fatahi B., Rasekh H. (2024). Stiffness and damping characteristics of jointed rocks under cyclic triaxial loading subjected to prolonged cyclic loading. Int. J. Fatigue.

[31] Zhou Z., Bai R., Shen M., Wang Q. (2022). The effect of overconsolidation on monotonic and cyclic behaviours of frozen subgrade soil. Transp. Geotech..

[32] Li P., Wu Y.F. (2016). Stress–strain behavior of actively and passively confined concrete under cyclic axial load. Compos. Struct..

[33] Shi D., Chen X., Ning Y., Ji T. (2023). Deformation responses and strength criteria of different shotcrete-rock composites under triaxial cyclic compression. Constr. Build. Mater..

[34] Fu Q., Bu M., Su L., Guo B., Chen L., Song H., Yuan Q., Niu D. (2021). Dynamic triaxial compressive response and failure mechanism of basalt fibre-reinforced coral concrete. Int. J. Impact Eng..

[35] Abood A.S., Fattah M.Y., Al-Adili A. (2023). Effect of saturation on dynamic characteristics of collapsible gypseous soil using cyclic triaxial testing. Case Stud. Constr. Mater..

